# Need for focus on microbial species following ice melt and changing freshwater regimes in a Janus Arctic Gateway

**DOI:** 10.1038/s41598-018-27705-6

**Published:** 2018-06-20

**Authors:** Nathalie Joli, Michel Gosselin, Mathieu Ardyna, Marcel Babin, Deo Florence Onda, Jean-Éric Tremblay, Connie Lovejoy

**Affiliations:** 1 Département de biologie, Québec Océan and Takuvik Joint International Laboratory (UMI 3376), Université Laval (Canada) - CNRS (France), Université Laval, Québec, QC G1V 0A6 Canada; 2 Institut de biologie intégrative et des systèmes (IBIS), Université Laval, Québec, QC GIV 0A6 Canada; 3 Institut des sciences de la mer de Rimouski, Université du Québec à Rimouski, 310 Allée des Ursulines, Rimouski, QC G5L 3A1 Canada; 4 Sorbonne Universités, UPMC Paris 06, INSU-CNRS, Laboratoire d’Océanographie de Villefranche, 181 Chemin du Lazaret, 06230 Villefranche-sur-mer, France; 5 Department of Earth System Science, Stanford University, Stanford, CA 94305 USA

## Abstract

Oceanic gateways are sensitive to climate driven processes. By connecting oceans, they have a global influence on marine biological production and biogeochemical cycles. The furthest north of these gateways is Nares Strait at the top of the North Water between Greenland and Ellesmere Island (Canada). This gateway is globally beneficial, first by supporting high local mammal and bird populations and second with the outflow of phosphate-rich Arctic waters fueling the North Atlantic spring bloom. Both sides of the North Water are hydrologically distinct with counter currents that make this Arctic portal a Janus gateway, after Janus, the Roman god of duality. We examined oceanographic properties and differences in phytoplankton and other protist communities from the eastern and western sides of the North Water (latitude 76.5°N) and found that species differed markedly due to salinity stratification regimes and local hydrography. Typical Arctic communities were associated with south flowing currents along the Canadian side, while potentially noxious *Pseudo-nitzschia* spp. were dominant on the Greenland side and associated with greater surface freshening from ice melt. This susceptibility of the Greenland side to *Pseudo-nitzschia* spp. blooms suggest that monitoring species responses to climate mediated changes is needed.

## Introduction

**Figure 1:**
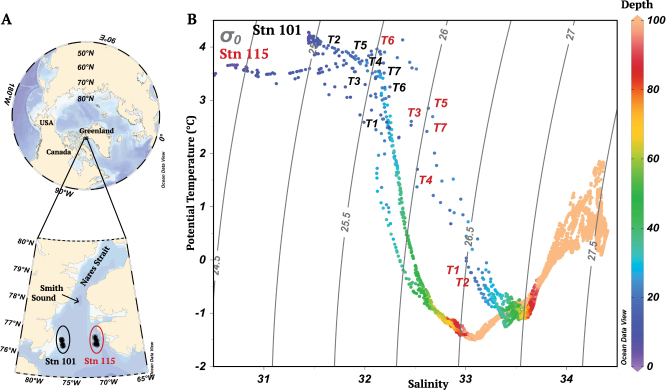
North Water sampling sites and temperature-salinity plots at the Canadian (Stn 101) and Greenland (Stn 115) repeated sampling sites. (**A**) Dots on the map corresponds to the drift sites where samples were collected (see Supplementary Table S1). (**B**) Potential temperature – salinity (T–S) diagrams from CTD profiles to 100 m. The T–S points are color coded for sampling depth. The 20 m samples are indicated by sequential sampling time (T_1_–T_7_) from the Canadian (black labels) and Greenland (red labels) sides.

Oceanic gateways have been critical in mediating past climatic changes and modulate heat and salinity exchange between ocean basins^1^. Nutrients and living organisms are also transported across these gateways and can influence local and global biogeochemical cycles^2,3^. The Arctic Ocean and surrounding seas are under threat due to rapid climate change^4^ with effects on net primary production and carbon fluxes^5,6^. The gateways to Baffin Bay (Nares Strait, Jones Sound and Lancaster Sound) between Canada and Greenland are of special interest as net Arctic outflows. These outflows influence nutrient balances^7,8^ and species composition in the North Atlantic^9^. Nares Strait is the deepest and the most northerly of all Arctic gateways and is contiguous with the historically productive North Water (locally referred to as Pikialasorsuaq), where human settlement dates to up to 1000 years before present (BP)^10^. The North Water Polynya (open water surrounded by sea ice) usually occurs in winter due to the formation of an ice bridge across Nares Strait^11,12^. The area supports large populations of marine mammals and seabirds^13^ partly due to a longer open water season enabling early season access to resources by migrating species, but also aided by estuarine type circulation that results in high productivity^14^. A northward flow along the west coast of Greenland (West Greenland Current) transports both surface and deeper warmer saltier nitrate-rich Atlantic Water as far north as Smith Sound at the southern end of Nares Strait (Fig. 1A), before being forced south, driven by cyclonic circulation^15^. As well on the Greenland side, non-sea ice sourced freshwater has been detected in the Polar Mixed Layer (PML) that lies between ca. 5 m and the first major halocline in the Arctic water column^16^. On the Canadian side of the North Water, Arctic Ocean water flows directly southward^12^, transporting cold phosphate rich water from the Central Arctic and Lincoln Sea, along with first year and multi-year sea-ice into the North Atlantic^17^.

Data from satellites indicate that the timing and extent of the spring–summer surface chlorophyll signal in the North Water region is highly variable but tending to occur further North over time^18^. While valuable, synoptic views from space lack information on chlorophyll concentrations below the surface^19,20^, and are blind to most species changes. Recent increasing northern penetration of Atlantic Water along the West Coast of Greenland and melting of the Greenland Ice Sheet (GrIS) could alter the nature of the Nares Strait gateway at multiple levels. For example, local assemblages of the eukaryotic phytoplankton and other protists likely have differing carbon and nitrogen cycle regimes^21^. In addition, since microbial eukaryotes are the base of the food chain, species changes could influence higher trophic levels^13^. Given sensitivity of the Arctic to climate change and the key role of Northern Baffin Bay in the Arctic-Atlantic system, an understanding of the species variability within this gateway is needed. An earlier microscopy study based on samples collected from a four-month series of transects from April to July 1998 in the North Water, found that microbial eukaryotic communities in both the surface and the upper waters that make up the Polar Mixed Layer (PML), differed regionally^22^. Since the Canadian and Greenland sides of the North Water region are influenced by different hydrological processes, our working hypothesis was that the microbial eukaryotic communities would differ in August, despite similar light levels at 20 m and the post bloom low nutrient conditions that follow spring and early summer high productivity and nutrient depletion^18,23^. The repeated transects of the 1998 study^24^ did not resolve short–term variability, in space or time, and the lack of closely spaced replicates, could be considered a shortcoming. Multiple profiles and sample collection at the same location is extremely rare in this region of the Arctic and one profile per station is routine^18^. In addition, previous expeditions to the North Water have sampled stations at more or less random times over the 24-hour cycle due to logistic constraints on multidisciplinary missions^25^. For these reasons, we repeatedly sampled the bottom of the PML in two one–day Lagrangian drifts, with one each of the Canadian and Greenland sides of the North Water (Fig. 1A, Supplementary Table S1). We spaced the sample collection approximately every 4 hours over 24 hours to account for any differences between the two sides that may have been associated with solar or tidal cycles. To investigate microbial eukaryotic community variability, we focused on the V4 region of 18 S rRNA using high throughput sequencing (HTS). Given that microbial eukaryotes are sensitive indicators of water masses^26^, the aim was to capture short term temporal and spatial variability in biological, chemical and physical parameters and link these to species composition and community changes.

## Results

### Oceanographic characteristics

**Figure 2:**
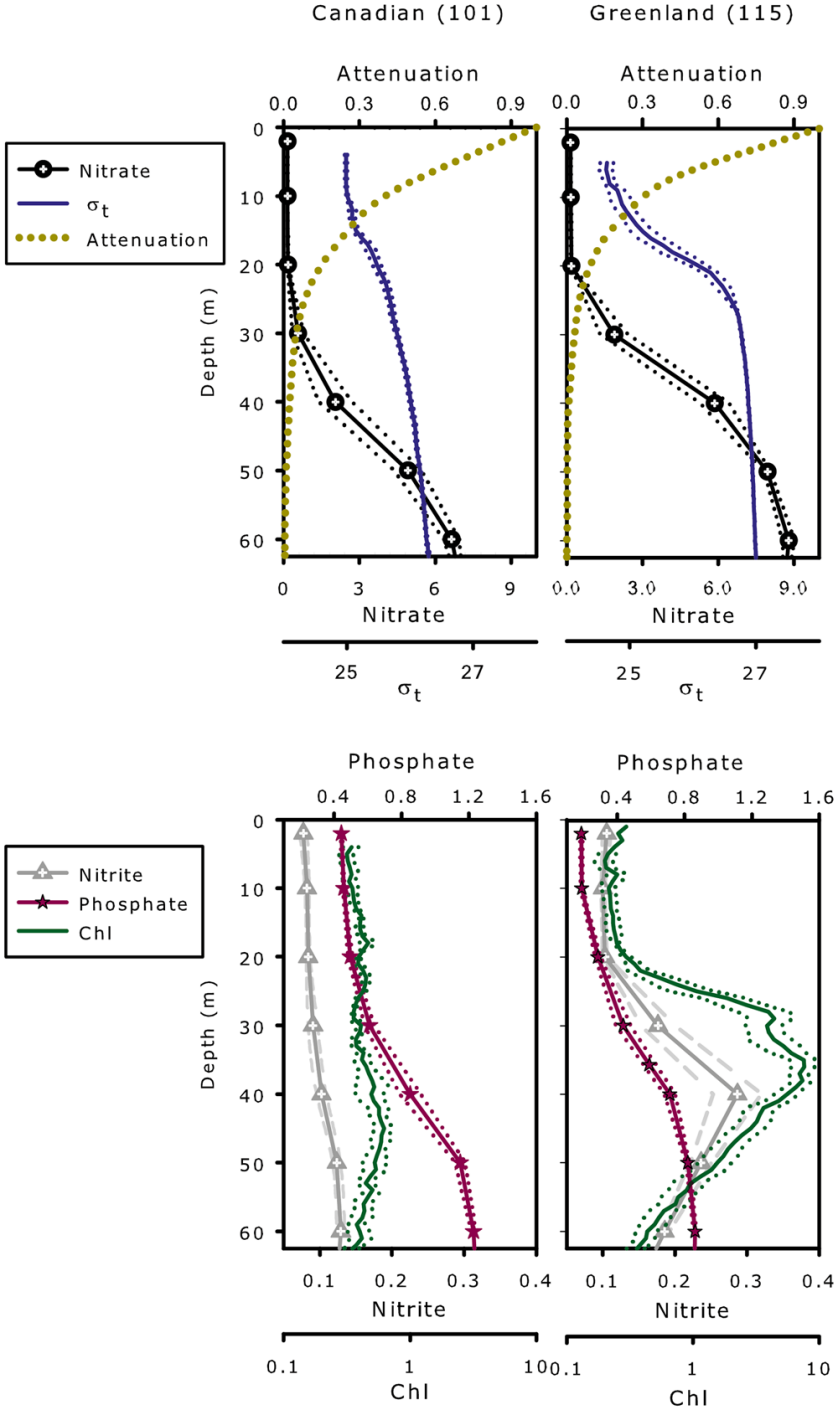
Vertical profiles of the upper 60 m of the water column on the Canadian side (Stn 101) and Greenland side (Stn 115) of the North Water. Upper panels) attenuation of photosynthetically available radiation (PAR), nitrate concentration (in µM), and water density (σ_*t*_ in kg m^−3^). Lower panels) phosphate and nitrite concentrations (in µM), and chlorophyll fluorescence (Chl in arbitrary units). The single dotted line for PAR attenuation was from T_2_ for Stn 101 and from T_3_ for Stn 115. Central solid lines are the average of 7 profiles (time T_1_ to T_7_), with exterior dotted lines showing the standard error.

At the time of sampling in mid-august, the temperature-salinity (TS) profiles showed fresher surface waters and more saline deep waters on the Greenland side compared to the Canadian side (Fig. 1B). On the Canadian side, there was a slight increase in salinity between the surface and the weak subsurface chlorophyll maximum (SCM) (31.45 to 32.35). This was in contrast to fresher surface waters (salinity of 30.51) and a sharp halocline on the Greenland side with salinity of 33.32 at the SCM (Table 1, Fig. 1B). The surface chlorophyll (Chl *a*) concentrations from satellite data were similar on the two sides and both sides had been ice free since the end of June (Supplementary Fig. S1). Total Chl *a* from samples collected at the surface were also similar on the two sides (Supplementary Fig. S2; ANOVA, p-value = 0.304). At the bottom of the PML starting at 20 m depth, the two sides differed in nearly all respects. On the Canadian side, *in situ* chlorophyll fluorescence (Fig. 2, upper pannel) and total Chl *a* concentrations (Supplementary Fig. S2A) changed little down the water column, with only a very weak SCM (0.69 mg Chl *a* m^−3^) detected at ca. 40 m. On the Greenland side, the SCM at 30 m was pronounced with concentrations reaching 2.56 mg Chl *a* m^−3^. Dissolved oxygen (DO) concentrations were also greater on the Greenland side (Table 1 and Supplementary Table S2) compared to the Canadian side. For nutrients, phosphate concentrations were higher down the water column on the Canadian side. Nitrate concentrations were depleted to at least 20 m on both sides and increased with depth below the PML with higher concentrations on the Greenland side. There was a nitrite maximum detected on the Greenland side at 40 m (Fig. 2).

**Table 1:** Environmental data for T_1_ to T_7_ from 20 m and the surface (Surf) and subsurface chlorophyll maxima (SCM) at T_1_ for Stn 101 and at T_2_ for Stn 115; Temperature (T) in °C, salinity (S_P_), dissolved oxygen (DO) in µM, relative fluorescence of colored dissolved organic matter (*f*CDOM), nitrite (NO_2_), nitrate (NO_3_), phosphate (PO_4_) and silicate (Si) in µM. Ration of silicate to nitrite + nitrate (Si:N).

Stn	Depth	Sample	T	S_P_	DO	*f*CDOM	NO_2_	NO_3_	PO_4_	Si	Si:N
101	20	T_1_	2.65	32.18	353.3	3.13	0.08	0.15	0.56	0.58	2.52
	20	T_2_	3.97	31.70	341.6	2.84	0.07	0.12	0.47	0.52	2.71
	20	T_3_	3.44	32.11	350.1	3.19	0.09	0.15	0.49	0.93	3.94
	20	T_4_	3.65	32.01	341.2	2.99	0.08	0.14	0.48	0.89	4.13
	20	T_5_	3.90	31.88	343.9	2.99	0.09	0.14	0.47	0.95	4.32
	20	T_6_	3.24	32.20	332.7	3.19	0.09	0.20	0.48	0.97	3.41
	20	T_7_	3.55	32.14	334.1	3.20	0.09	0.28	0.53	0.93	2.50
	2	Surf	4.27	31.45	336.7	2.98	0.08	0.21	0.46	0.61	2.09
	38	SCM	1.33	32.35	352.8	3.79	0.10	0.72	0.71	0.33	0.41
115	20	T_1_	−0.13	33.03	414.4	2.67	0.11	0.21	0.33	0.66	2.09
	20	T_2_	−0.41	33.11	410.9	3.02	0.10	0.18	0.32	0.52	1.87
	20	T_3_	2.60	32.46	374.3	2.41	0.11	0.19	0.32	1.00	3.35
	20	T_4_	1.37	32.52	373.8	2.18	0.09	0.26	0.23	1.10	3.08
	20	T_5_	2.85	32.63	367.1	2.26	0.10	0.18	0.30	0.31	1.14
	20	T_6_	3.90	32.20	317.1	2.19	0.11	0.12	0.26	0.72	3.20
	20	T_7_	2.42	32.61	361.3	2.20	0.11	0.15	0.24	0.80	3.02
	2	Surf	3.47	30.51	332.3	2.19	0.11	0.20	0.20	1.30	4.25
	30	SCM	−0.91	33.32	333.6	3.63	0.27	3.97	0.61	4.26	1.01

Our repeated sampling indicated that nitrate and silicate concentrations at 20 m were similar on both sides of the North Water (Supplementary Table S2). However, phosphate concentrations and fluorescent colored dissolved organic matter (*f*CDOM) showed significant differences between the two sides at 20 m. On the Greenland side, phosphate (avg. 0.29 µM) and *f*CDOM (avg. 2.42 fluorescence units) were significantly lower compared to the Canadian side (phosphate avg. 0.49 µM and *f*CDOM avg. 3.08 units) (Table 1, Supplementary Table S2).

### Microbial communities

**Figure 3:**
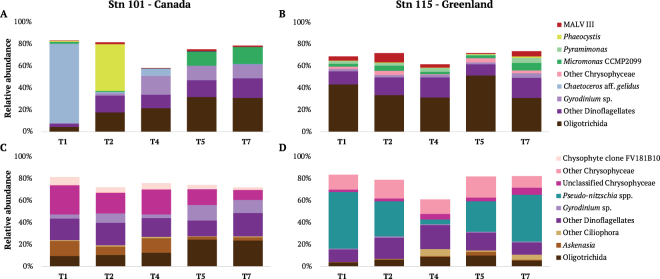
Barplot of the most abundant species per station per fraction. (**A**) Stn 101 small fraction. (**B**) Stn 115 small fraction (**C**) Stn 101 large fraction. (**D**) Stn 115 large fraction.

**Figure 4:**
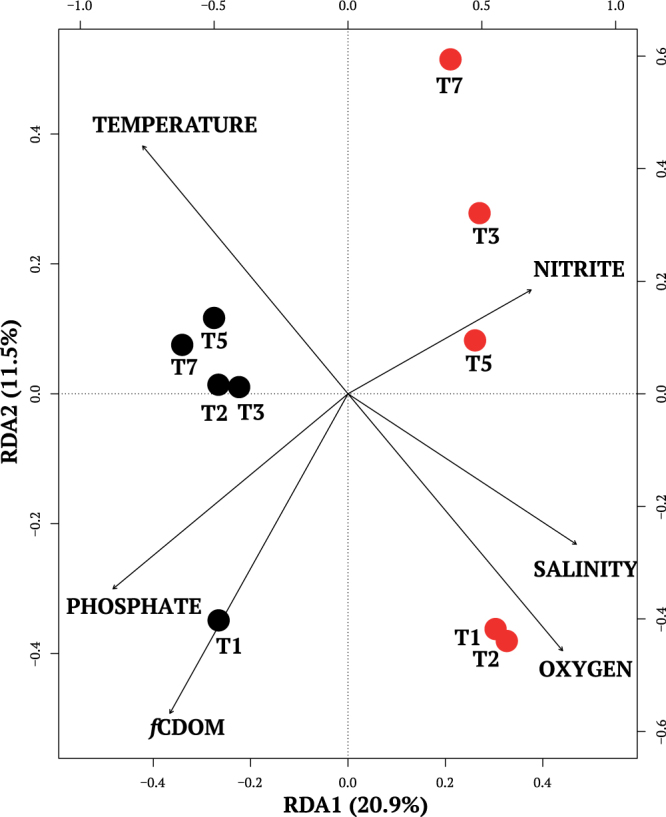
Redundancy analysis from (Canadian) Stn 101 (black dots) and (Greenland) Stn 115 (red dots) based on unweighted UNIFRAC values from the entire microbial eukaryote community within each sample.

For T_1_ and T_4_, both DNA and RNA templates were sequenced. The relative proportions of taxa inferred from the DNA (rDNA, Supplementary Fig. S3) was similar to proportions of the same taxa from the samples inferred from the RNA (rRNA, see bars for T_1_ and T_4_ in Fig. 3). A general exception was a higher proportions of dinoflagellates in the rDNA (Supplementary Fig. S3, Supplementary Table S3). Both templates showed the marked difference in the microbial eukaryote communities on the two sides of the North Water (Fig. 3, Supplementary Fig. S3). Unweighted clustering of the rRNA dominants Operational Taxonmic Units (OTUs with >100 read occurrences) also clearly separated communities on the two sides (Supplementary Fig. S4). Environmental variables (Supplementary Table S2) that were significantly correlated with Principle Coordinates Analysis (PCoA) community values were used for the Redundancy Analysis (RDA). The Greenland and Canadian sides clearly separated along the RDA1 axis explaining 20.9% of the total variance (Fig. 4). Higher phosphate, *f*CDOM and temperature were associated with the Canadian communities, with nitrite, DO and salinity associated with the Greenland communities. Analysis of variance (ANOVA) repeated measures and Kruskal-Wallis tests supported the RDA results (Supplementary Table S2).

Over the ca. 24 hours of 20 m repeated sampling, the Canadian side communities varied substantially, with the T_1_ community predominantly the small diatom, *Chaetocero*s aff. *gelidus* (up to 72% of reads at T_1_). *Chaetoceros* had largely disappeared 4 hours later; replaced by *Phaeocystis*, which accounted for 43% of the small fraction reads at T_2_. Subsequently we detected a mix of small photosynthetic flagellates, which co-occurred with another Hylochaete *Chaetoceros* species; *C. neogracile* (Fig. 3, Supplementary Table S4A). The communities on the Greenland side at 20 m were less variable (Supplementary Table S5), with all samples containing the potentially toxic pennate diatom *Pseudo-nitzschia* (up to 52% of rRNA reads at T_1_). The two dominant diatom genera *Chaetoceros* and *Pseudo-nitzschia* were genetically diverse with multiple OTUs found on both the Canadian and Greenland sides (Supplementary Fig. S5). The presence of both genera was confirmed microscopically (Supplementary Fig. S6 plates a,b for *Chaetoceros*, and Fig. S6 plates g,h,i,j for *Pseudo-nitzschia*).

Alveolates represented the second most abundant major group from rRNA on both sides of the North Water. Ciliates made up 28% of the total reads on the Canadian side and 30% of the total reads on the Greenland side (Fig. 3, Supplementary Table S4). Most of the ciliate OTUs were in the order Oligotrichida, with contributions from Choreotrichida, Cyclotrichida (*Askenasia*), Prorodontida *(Urotricha*), Haptorida (*Monodinium*) and other unidentified Litostomatea detected on both sides. *Urotricha* was common in the large fraction on the Canadian side but less on the Greenland side. In contrast, the genus *Monodinium* was more abundant on the Greenland compared to the Canadian side (Supplementary Table S4). Core dinoflagellates accounted for ca. 30% of total reads on the Canadian side and 21% of total reads on the Greenland side. For both sides, unclassified dinoflagellates accounted for 12% to 18% of reads (Supplementary Table S4). The most common dinoflagellate OTUs on the two sides were Gymnodiniales. At the species level *Gyrodinium spirale* was common on the Canadian side and *Torodinium robustum*, which was also identified microscopically (Supplementary Fig. S6 plates c,d) was common on both sides. The majority of dinoflagellate taxa were morphologically diverse gymnodinoids and gyrodinoids between 6–50 µm and not identified further using microscopy. From HTS, Syndiniales were predominantly in the Duboscquellidae in Group I marine alveolates (MALV I)^27^ and the still poorly known environmental MALV III, associated with the environmental clone OLI11005^28^ (GenBank AJ402349).

The relative abundances of smaller flagellated taxa differed between the two sides. For example, several haptophyte OTUs were more common on the Canadian side with *Phaeocystis* accounting for 8.8% of reads in the small size fraction, and *Chrysochromulina* with 1.2% in the large fraction (Fig. 3, Supplementary Table S4). The Arctic *Micromonas* CCMP 2099 was relatively more abundant on the Canadian side with 6.1% of the small fraction reads, compared to the Greenland side with 3.8% of the reads (Fig. 3, Supplementary Table S4). A second chlorophyte, *Pyramimonas* sp. (Supplementary Fig. S6 plates i,j), accounted for nearly 3% of small fraction reads on the Greenland but only 0.1% on the Canadian side. Two heterotrophic flagellate taxa; Picozoa and group 2 uncultivated marine stramenopiles (MAST 2), were common on the Greenland side with each accounting for 1.9% of reads in the small fraction. There were marked differences in the dominant chrysophytes on the two sides (Fig. 3 and Supplementary Table S4) with *Dinobryon balticum* identified from the Canadian side (Supplementary Fig. S6, plates e,f) and *D. belgica* on the Greenland side (Supplementary Fig. S6 plates k,l).

## Discussion

Within the PML the microbial eukaryotic communities from the two sides of the North Water differed taxonomically, with *Pseudo-nitzschia* dominated communities persistently found on the Greenland side, and more mixed communities on the Canadian side. The Canadian communities changed several times over the nearly 24 hours of sampling (Fig. 3). The high similarity among samples collected on the Greenland side (Supplementary Table S5) suggested that a single sample could have been used to represent the community in mid-August. In contrast, no single typical community was detected on the Canadian side. The active communities detected using rRNA, were consistent with the physical oceanography of the respective sides. Salinity determines Arctic Ocean physical oceanographic structure^29^ and on the Greenland side, Atlantic waters contribute to the higher salinity and sensible heat at depth^11^, which leads to shoaling of the upper mixed layer^30^. During our study the fresh surface waters on the Greenland side originated from first year and multi-year sea ice melt and also likely from iceberg and glacier melt from around Greenland^16,31^. A high external input of surface fresh water originating from the Greenland Ice Sheet (GrIS) was previously suggested as an explanation for anomalous values for alkalinity and *delta* O^18^ data from the same station on the same cruise^16^. The Canadian side reflected the more general freshening of the Arctic Ocean that is due to multi-year sea ice melt^32^. This overall freshening on the Canadian side could be seen in the weak halocline and lower average water column salinity (Fig. 1B, Fig. 2). The higher phosphate and *f*CDOM concentrations (Table 1, Table S2), on the Canadian side were also characteristic of Arctic Water^33^, in keeping with the Nares Strait Gateway directly contributing excess Arctic Ocean phosphate to the Western Labrador Sea and North Atlantic^7,8^.

At the surface, the two sides of Northern Baffin Bay were similar in terms of ice cover and surface bloom phenology (Supplementary Fig. S1), with surface chlorophyll concentrations on both sides within the long-term norm for mid-August^18^. However, below the surface, along with the marked differences in physical structure, differences in biological indicators, such as the prominent SCM and overall higher Chl *a* concentrations at depth on the Greenland side, were striking. The formation of the Arctic SCM below the PML depends on the availability of both light and nutrients^34^ and the high nitrate concentrations below 30 m on the Canadian side, suggest that phytoplankton may have been light limited at that depth. If so, light limitation could be explained by water column instability associated with mixing below the halocline, moving phytoplankton out of the euphotic zone to depths below irradiance levels sufficient for net growth. Alternatively, or in addition, the lack of formation of a prominent SCM on the Canadian side may have been due to the hydrologic complexity of the region, where multiple water-masses with similar densities and different recent histories could converge and interleave^35^. Such interleaving would be consistent with temperature-salinity (TS) profiles (Fig. 1B) and rapid community changes (Fig. 3) at the single depth sampled. In contrast, there was a prominent SCM on the Greenland side, which was associated with a well stratified water column and presumably continuous nutrient supply to the bottom of the euphotic zone beneath the sharp halocline. Saline Atlantic water below the fresher PML is a characteristic of the Greenland side^30^, and such ideal conditions for SCM formation^36^ (Fig. 2, Supplementary Fig. S2) would be expected, suggesting annual formation of an SCM on the Greenland side.

The RDA (Fig. 4) supported the notion that the distinct communities on the two sides were associated with the physical oceanography of the region^17^. Higher phosphate and *f*CDOM concentrations, characteristic of Arctic surface waters^23^ were associated with samples collected from the Canadian side. In contrast, Greenland side communities were associated with greater DO concentrations consistent with local upward diffusion of DO from the photosynthetic activity at the much more robust SCM.

The active community from the RNA template (Fig. 3, Supplementary Table S4), documented by microscopy (Supplementary Fig. S6), revealed the presence of Hyalochaete *Chaetoceros* predominantly at T_1_ on the Canadian side, when the colder temperatures and the high *f*CDOM indicated the highest contribution of unmodified Arctic waters^33^. These small diatoms were earlier reported (identified as *C. socialis*) as ubiquitous in the summer phytoplankton in the North Water^24^. The abundant *Chaetoceros* populations suggested that the 20 m sample at T_1_ may have originated nearer the surface under high irradiances^37^. At T_2_ on the Canadian side, the community changed, with a high proportion of reads from *Phaeocystis*, which is another common polar species. For example, *Phaeocystis* is reported from the Ross Sea, Antarctica^38^. Several studies suggest that *Phaeocystis* rapidly responds to increased light and nutrient inputs^39,40^ consistent with changing conditions on the Canadian side of the Nares Strait gateway. Another small flagellate *Micromonas polaris*, which was recently described as an endemic arctic species^41^, and almost universally reported from Arctic 18 S rRNA surveys as *Micromonas* CCMP 2099^42,43^, was retrieved on both sides but with highest relative abundance after T_5_ on the Canadian side (ca 14% in the small fraction; Fig. 3). Other typical Arctic flora on the Canadian side included chrysophytes such as *Dinobryon balticum*, which contributes to carbon flux in cold waters^44^. In contrast, the morphospecies *Dinobryon belgica*, which has smaller colonies (Supplementary Fig. S6 plate e versus l) was noted on the Greenland side. The 30-fold difference in the abundance of *Pyramimonas* on the Greenland versus Canadian side may have been facilitated by the low salinity surface waters. For example, Daugbjerg^45^ indicated that some *Pyramimonas* species are able to tolerate a wide range of salinity and *Pyramimonas* was also reported as a dominant nanoflagellate in surface waters with high freshwater input from the Mackenzie River and surface ice melt in the Beaufort Sea^43^. In addition, *Pyramimonas* is associated with meltwater pools on sea ice^46^ and found during the winter-spring transition in the fjord and ice influenced Disko Bay in Greenland south of our study region^47^.

The molecular data (Fig. 3, Supplementary Fig. S3, Supplementary Table S3, Supplementary Table S4) verified using microscopy (Supplementary Fig. S6) showed that a major difference between the two sides was the overwhelming dominance of *Pseudo-nitzschia* spp. on the Greenland side, accounting up to 52% of reads, but <0.1% of reads on the Canadian side. The presence of *Pseudo-nitzschia* is of concern, since the genus can produce the neurological toxin, domoic acid (DA)^48^. In contrast, more typical summer Arctic communities^49^ were found on Canadian side, with variability consistent with changes in water masses with different recent histories and species^35^. *Pseudo-nitzschia* has long been present in this region, but high concentrations have not been previously reported. During the 1998 North Water Polynya (NOW) study, Lovejoy *et al*.^22^ observed a diverse diatom community on the Greenland side in July, including *Pseudo-nitzschia* cf. *seriata*. A *Pseudo-nitzschia* strain (CCMP 2093) now classified as *P. arctica*^50^ was isolated from the North Water (78°35.87′N, 74°29.53′W) in 1998 and other *Pseudo-nitzschia* species have been isolated from Greenland fjords south of our study region^51^. *Pseudo-nitzschia* have wide salinity tolerances, are able to use a variety of nitrogen sources, and are favored under conditions of high dissolved Fe concentrations^52^. The nitrite peak at 40 m on the Greenland side suggests high biological activity and an additional nitrogen source for *Pseudo-nitzschia*^53^. Overall, we suggest that GrIS inflows could favor blooms of *Pseudo-nitzschia* spp. and potentially DA production^48^. DA producing *Pseudo-nitzschia* blooms are detrimental to marine ecosystems since DA causes memory impairment and is harmful to marine mammals^54^. To date, DA has not been reported from the North Water, but there have been few or no DA surveys in the region, to our knowledge. Locally isolated *Pseudo-nitzschia* strains from Disko Bay (Greenland) produce DA when fed to Arctic copepodites^51^ and *Pseudo-nitzschia* OTUs here clustered with species such as *P. brasiliana* (Supplementary Fig. S5A), which is known to produce DA^52^, suggesting the potential of DA production. Recently, large-scale phytoplankton blooms in the Labrador Sea were reported to coincide with freshwater discharge from glaciers and meltwater from the GrIS, however information on species was lacking^55^, and given that the bloom was attributed to increased Fe and organic nutrients, it would not be surprising if *Pseudo-nitzschia* was present, and such phenomena warrant investigation.

In sum, although classified as a single eco-region for modeling studies^56^, The Greenland side of the gateway was more saline below PML, but fresher on the surface (Fig. 1B), consistent with recent increases in melt water from the Greenland Ice Sheet (GrIS)^57^. Freshwater from the GrIS, glacial melt and multi-year sea-ice melt enter the North Water via Nares Strait as surface water along the Greenland side^17^. Additional GrIS melt from Northeast Greenland^58^ could also contribute to surface freshwater on the Greenland side of the North Water since it flows southward along the east coast of Greenland (via the East Greenland Current) and at the southern tip of Greenland, becomes entrained into the northward coastal flow (West Greenland Current)^57^. Any increase in GrIS meltwater and associated high iron (Fe) and other micronutrient concentrations^59^ has implications for the biogeochemistry of Baffin Bay^55^ and would create conditions that could favor blooms of *Pseudo-nitzschia*. This in turn, suggests a threat to the historic role of the North Water in supporting marine bird and mammal populations including endangered Greenland populations of Bowhead whales, which migrate to this latitude in summer and graze on copepods^60^. Finally, given the increasing cyclonic circulation in Northern Baffin Bay, low-phosphate waters and potential toxic species might flow south to the North Atlantic along the Baffin Bay meridional ridge^17^, making this a true Janus gateway. This work highlights the need to consider drivers that select for species and the biological diversity of gateways, if we are to fully understand impacts of climate change in the Arctic and elsewhere.

## Materials and Methods

### Satellite-derived products

Satellite-derived level-3 data sets of Chl *a* concentration (mg m^−3^) and particulate back-scattering coefficients at 443 nm (b_bp_, m^−1^) (Supplementary Fig. S1) were obtained from the European Space Agency’s GlobColour project (http://www.globcolour.info). Eight-day composite Chl *a* concentration and b_bp_ coefficients were determined, respectively using standard Case 1 water algorithms^61^ and the semi-analytical Garver Siegel Maritorena (GSM) merging algorithm^62^. The sea-ice concentration was derived from the Advanced Microwave Scanning Radiometer - Earth Observing System (AMSR-E) sensor, and made available by National Snow and Ice Data Center (NSIDC; https://nsidc.org). Actual sea ice conditions northward and just prior to sampling were further verified by inspecting a range of additional satellite products (https://worldview.earthdata.nasa.gov/).

### Field sampling

Oceanographic data was collected aboard the CCGS *Amundsen* using a rosette system equipped with a conductivity, temperature, depth (CTD) profiler (Sea-Bird SBE-911 CTD), relative nitrate (*In-Situ* Ultraviolet Spectrometer, ISUS, Satlantic), oxygen (Seabird SBE-43), chlorophyll fluorescence (Seapoint), fluorescent colored dissolved organic matter (*f*CDOM; Wetlabs ECO) and photosynthetically available radiation (PAR, 400–700 nm; Biospherical Instruments QDP2300) sensors. The oxygen sensor was calibrated onboard against Winkler titrations^36^.

The ship followed a buoy with a drogue suspended to 20 m, with the goal of following the bottom of the PML and sampled approximately every 4 hours beginning at solar time 05:15 for Stn 101 (Canadian side) and 06:00 for Stn 115 (Greenland side), ending after 23 hours at and 22.5 hours, respectively (Supplementary Table S1**)**. At Stn 101, the drift started (T_1_) at lat 76°23.242′N, long 077°23.412′ W, and ended (T_7_) at lat 76°17.372′N, long 077°46.264′W. The Stn 115 drift started (T_1_) at lat 76°20.297′N, long 071°11.514′W and ended at (T_7_) lat 76°29.858′N, long 071°26.615′W (Fig. 1A and Supplementary Table S1). Water samples were collected on the upcast from 12–L Niskin-type bottles mounted on the rosette. Nutrient samples were collected every 10 m from the surface to 60 m. Nutrients were analyzed on board within 4 h after collection as using a Bran-Luebbe 3 autoanalyzer^63^. Optical depths were derived from vertical PAR profiles taken from the shadow-free side of the ship using a PNF-300 radiometer (Biospherical Instruments). At time T_1_ for Stn 101 and time T_3_ for Stn 115, water samples for duplicate Chl *a* were collected at seven optical depths (100, 50, 30, 15, 5, 1, and 0.2% of surface irradiance), the SCM determined from the downward cast of the CTD, and at 80 m and 100 m. Chl *a* samples were also collected and analyzed from 20 m along with nucleic acids (see below) during the drift. Samples for total Chl *a* were filtered onto 25 mm Whatman GF/F filters (TChl *a*; >0.7 µm) and onto 5 µm pore size polycarbonate (PC) membrane filters (Nuclepore™) to estimate the large fraction (L-Chl *a*; >5 µm) and analyzed on board as in Parsons *et al*.^64^. Concentrations in the smaller fraction (S-Chl *a*; 0.7–5 μm) were from subtraction. For nucleic acids, five independent samples from 20 m were collected from each the Canadian and Greenland sides (Supplementary Table S1). Six L of sample water prefiltered through a 50 μm nylon mesh, and then sequentially filteredthrough a 47–mm diameter 3 μm pore size PC membrane filter, and a 0.2 μm Sterivex™ Unit (Millipore). Material on the filters was preserved in RNAlater™ (ThermoFisher) then frozen at −80 °C. The different size fractionation protocols for Chl *a* and nucleic acids was due to logistic constraints aboard the ship, and small and large designations should be considered as indicative only.

Whole water samples for fluorescence microscopy were collected at 20 m at T_1_ for Stn 101 and T_2_ for Stn 115. Single aliquots of 48 mL were preserved and filtered onto black 0.8 µm PC filters, stained with 4,6-diamidino-2-phenylindole (DAPI) following^65^. The slides were inspected at 1000X magnification using an Olympus IX71 microscope equipped with UV and blue excitation filter blocks. Images were captured with the integrated QImaging Retiga 2000R CCD Camera and QCapture software version 2.9.11.

### Laboratory procedures

DNA and RNA were extracted from the same filters using the All-Prep DNA/RNA Minikit (Qiagen). DNA was used to verify the overall community including dead and dormant cells at two time points 12 hours apart on either side (T_1_ and T_4_). RNA was converted to cDNA using the High Capacity Reverse Transcription Kit (Applied Biosystems). The V4 region of 18 S rRNA gene and rRNA (from cDNA) was amplified using the eukaryote specific primers E572 (forward) and E1009 (reverse), see Comeau *et al*.^66^, coupled with a MiSeq© specific linking primer. To decrease potential PCR bias, 1, 5 and 10-fold diluted template was used for PCRs for each sample. The PCR products of the three dilutions were pooled together and purified using the Axygen® PCR cleanup kit (Axygen) and then quantified spectrophotometrically with the Nanodrop 1000™ (ThermoFisher Scientific). Unique pairs of barcodes (tags) were added to the sample amplicons using the TruSeq® and Nextera® (both Illumina) barcode sets in a nested PCR as described in Comeau *et al*.^67^. Equimolar concentrations of the sample barcoded amplicons were sequenced on the Illumina MiSeq at the Plate–forme d’Analyses Génomiques (IBIS, Université Laval, Québec, QC, Canada). All reads are deposited in NCBI GenBank Sequence Read Archive (SRA) under the BioProject number PRJNA383398 (GenBank: SRX2745611 to SRX2745642).

### Data and statistical analysis

Paired end reads were processed with UPARSE^68^, with microbial eukaryote operational taxonomic units OTUs retained following Wu *et al*.^69^. Based on a 99% similarity the reads clustered into 2069 OTUs. We then used an arctic specific reference database for taxonomic assignment of OTUs^70^. The resulting OTU classifications are available at https://zenodo.org/record/1205261#.WrOAMJPwb-Y and their associated reads at https://zenodo.org/record/1205255#.WrN_RJPwb-Y.

Data sets were further processed in QIIME^71^. Rarefied datasets (11,500 sequences per sample) were used as input for the UniFrac unweighted distances^72^, with beta diversity and principal coordinates (PCoA) as output. Environmental variables that were significantly correlated with PCoA community values were used for the redundancy analysis (RDA)^73^ computed using the *rda* function in the Rstudio package ‘vegan’ (v2.4-1)^74^. Parameters that best explained variability in the PCoA were selected using the ordistep function in the ‘vegan’ package to build an optimal model (highest adjusted coefficient determination). One-way ANOVA (parametric) and Kruskal-Wallis (non-parametric) tests were carried out in PAST v3.0^75^ to test for differences between the two sides of the North Water. ANOVA repeated measures tests were carried out using PAST v3.0^75^. Statistical tests of physical and nutrient data and profile plots were carried out in Sigma Plot (v11).

With the aim to focus on more abundant organisms, we selected OTUs that had a minimum of 100 occurrences in at least one rRNA sample from both sides of the North Water Clusters and heatmaps were constructed using the heatmap.2 function in gplots package in R. Rarefied rRNA read counts were first log transformed and then clustered by site (Y axis) and by OTUs (X axis) using Euclidean distances. Dendograms were generated using hierarchical clustering UPGMA.

To explore the diversity of the two dominant diatom genera in our samples, reference sequences related to *Pseudo-nitzschia* were retrieved from Percopo *et al*.^50^ and those related to *Chaetoceros* were retrieved from Chamnansinp *et al*.^76^. The V4 18 S rRNA sequences were aligned separately with MAFFT (v7)^77^.

## Electronic supplementary material

Supplementary material
