# Supplementary material for: Need for focus on microbial species following ice melt and changing freshwater regimes in a Janus Arctic Gateway
Source: Sci Rep. 2018 Jun 20;8:9405. doi: 10.1038/s41598-018-27705-6 (PMC6010473; doi:10.1038/s41598-018-27705-6)
Supplement: Supplementary file 1 — Supplementary material [file 41598_2018_27705_MOESM1_ESM.docx]

**Supplementary material on line:**

**Need for focus on microbial species following ice melt and changing freshwater regimes in a Janus Arctic Gateway.**

Nathalie Joli^a,b*^, Michel Gosselin^c^, Mathieu Ardyna^d,e^, Marcel Babin^a^, Deo Florence Onda^a,b^, Jean-Éric Tremblay^a^, Connie Lovejoy^a,b^

^a^Département de biologie, Québec Océan and Takuvik Joint International Laboratory (UMI 3376), Université Laval (Canada) - CNRS (France), Université Laval, Québec QC G1V 0A6, Canada

^b^Institut de biologie intégrative et des systèmes (IBIS), Université Laval, Québec, QC GIV 0A6, Canada

^c^Institut des sciences de la mer de Rimouski, Université du Québec à Rimouski, 310 Allée des Ursulines, Rimouski, Québec G5L 3A1, Canada

^d^Sorbonne Universités, UPMC Paris 06, INSU-CNRS, Laboratoire d'Océanographie de Villefranche, 181 Chemin du Lazaret, 06230 Villefranche-sur-mer, France

^e^Department of Earth System Science, Stanford University, Stanford, CA, 94305, USA

*Corresponding author email address: [joli@biologie.ens.fr](mailto:joli@biologie.ens.fr)

*Current address: Institut de Biologie de l'École Normale Supérieure (IBENS), CNRS UMR 8197, 46 rue d'Ulm 75005 Paris, France

**Table S1:** Latitude, Longitude and other metadata of samples used in this study.

**Table S2:** Results of ANOVA repeated measures and Kruskal-Wallis tests comparing the paired 20 m samples**.**

**Table S3:** Relative abundance of taxa in small and large fractions based on OTUs at the lowest reliable taxonomic level with occurrences of >1% of the overall reads in at least one DNA sample. Values less than 0.1% indicated as a dash (-).

**Table S4:** Operational taxonomic Units (OTUs) with > 1% of the reads in at least one sample. Relative abundance of taxa in small and large fractions based on OTUs at the lowest reliable taxonomic level with occurrences of  >1% of the overall reads in at least one RNA sample. Values less than 0.1% indicated as a dash (-). Average and standard deviation (Avg (sd)) **A)** Canadian side Stn 101 **B)** Greenland side Stn 115.

**Table S5:** Bray-Curtis similarity of communities from the two sides of the North Water

**Figure S1:** Composite satellite data. Sea ice cover, chlorophyll *a* concentration and particulate back-scattering coefficient at 443 nm estimated from satellite data. Stn 101 (Canada, left) and Stn 115 (Greenland, right). Vertical dashed lines represent the date of sampling; 15 August 2013 for Stn 101 and 18 August 2013 for Stn 115. Shaded area is % sea ice cover.

**Figure S2**: Extracted chlorophyll from the two sides of the North water. **A)** Profiles of total Chl *a* from Canadian (Stn 101) collected at T_1_ and Greenland (Stn 115) collected at T_2_ (**Table S1**). **B)** Size fractionated Chl *a* concentrations: smaller fraction (S) from 0.8–5 µm, larger(L) fraction > 5 µm and total (T***)*.** Box plots (n = 7) of repeated sampling from 20 m at the two stations. Central line is the median; differences between the stations were not significant (ANOVA repeated measures).

**Figure S3**: Barplot of the most abundant species from the DNA samples collected at two time points (T_1_ and T_4_) on each of the Canada side (A and C) and of the Greenland side (B and D) of Northern Baffin Bay for small (A and B) and large (C and D) fractions.

**Figure S4**: Heatmap from the reads of OTUs from rRNA accounting for at least 100 occurrences in one RNA 20 m sample from both sides. OTUs are in columns, color coded for taxonomic groups. Samples labels indicate station, time collected and small (S, 0.2-3 µm), or large (L, 3-50 µm) fractions. Color code as for Figure S3. Identity of OTUs available at: <https://zenodo.org/record/1205261#.WrOAMJPwb-Y>.

**Figure S5**: V4 region alignments. Position 572nt to 1009nt of the 18S rRNA gene. Reference sequences, with GenBank accessions and individual OTUs found on the GREENLAND, CANADA or BOTH sides. For visual comfort, identical bases are masked **A)** *Pseudo-nitzschia* and *Cylindrothica* (the closest related genus). The break between the genera is between OTU2945 (*Cylindrotheca*) and OTU3085 (*Pseudo-nitzschia*). **B)** Hylochaete *Chaetoceros*.

**Figure S6**: Epifluorescence micrographs showing DAPI stained cells under blue (a,c,e,g,i,k) and UV (b,d,f,h,j,l) excitation. Images from samples collected at Stn 101 with (a,b) *Chaetoceros gelidus*, (c,d) *Torodinium robustum*, and (e,f) *Dinobryon balticum*. Images from samples collected at Stn 115 with (g,h) *Pseudo-nitzschia* spp., (i,j) *Pyramimonas*, and (k,l) *Dinobryon belgica*.

**Table S1**: Latitude, Longitude and other metadata of samples used in this study. Samples collected on 15 August 2013 for the Canadian (101 Cdn) and 18 August 2013 for the Greenland (115 Grl) sides of the North Water. 20 m was repeatedly sampled. The ship was following a drifter and the latitude (Lat), longitude (Long), time and depth in meters (m) of sample collection indicated. Time of collection is given in UTC and calculated solar time (Solar). Sequencing indicates the number of clean microbial eukaryote V4 reads from RNA converted to cDNA, for Small (0.2–3µm) and Large (50–3 µm) size fractions, a dash (–) indicates no data.

| **Stn** | **Depth** | **Sample** | **Lat** | **Long** | **Time** | | **Sequencing** | |
| --- | --- | --- | --- | --- | --- | --- | --- | --- |
|  | **m** |  | **(°N)** | **(°W)** | **UTC** | **Solar** | **Reads Large** | **Reads**  **Small** |
| **101** | 20 | T_1_ | 76.45 | 76.45 | 09:30 | 05:16 | 26506 | 17560 |
| Cdn | 20 | T_2_ | 76.61 | 76.61 | 13:25 | 09:10 | 18853 | 29758 |
|  | 20 | T_3_ | 76.37 | 76.37 | 17:52 | 13:37 | – | – |
|  | 20 | T_4_ | 76.60 | 76.60 | 17:33 | 17:18 | 14908 | 26046 |
|  | 20 | T_5_ | 76.57 | 76.57 | 01:47 | 21:32 | 31768 | 21116 |
|  | 20 | T_6_ | 76.46 | 76.46 | 05:47 | 01:32 | – | – |
|  | 20 | T_7_ | 76.39 | 76.39 | 08:20 | 04:04 | 34000 | 19709 |
| **115** | 20 | T_1_ | 76.42 | 71.33 | 09:48 | 05:59 | 21395 | 43655 |
| Grl | 20 | T_2_ | 76.47 | 71.44 | 13:20 | 09:30 | 30019 | 28393 |
|  | 20 | T_3_ | 76.58 | 71.37 | 16:50 | 13:00 | – | – |
|  | 20 | T_4_ | 76.47 | 71.41 | 20:35 | 16:45 | 21676 | 17753 |
|  | 20 | T_5_ | 76.45 | 71.65 | 01:33 | 21:43 | 26420 | 32887 |
|  | 20 | T_6_ | 76.65 | 71.58 | 05:15 | 01:25 | – | – |
|  | 20 | T_7_ | 76.72 | 71.60 | 08:15 | 04:25 | 45533 | 12794 |


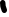


**Table S2:** Results of ANOVA repeated measures and Kruskal-Wallis tests comparing the paired 20 m samples**.** ANOVA repeated-measures (parametric) and Kruskal-Wallis (non-parametric) tests comparing paired 20 m samples (T_1_, T_2,_ T_4,_ T_5_ and T_7_) collected from the two sides of the North Water (Table S1). Variables tested: Temperature (T), Salinity (S), dissolved oxygen (DO), fluorescent colored dissolved organic material (*f*CDOM), nitrate (NO_3_), nitrite (NO_2_), dissolved silicon (Si), phosphate (PO_4_) and the Si:(NO_3_+NO_2_) ratio. Values are given in Table 1 of the main text. Direction of the statistically significant differences are noted (Conclusion), no significant difference (ns). Stn 101 (Canadian) and 115 (Greenland).

|  | **ANOVA** | | **Kruskal-Wallis** |  |
| --- | --- | --- | --- | --- |
| Variable | *F* | *p-value* | *p-value* | *Conclusion* |
| **T** | 11.11 | 0.01 | - | Stn 101 warmer |
| **S** | - | - | 0.009 | Stn 101 fresher |
| **DO** | 13.34 | 0.006 | - | Stn 115 higher O_2_ |
| ***f*CDOM** | 10.12 | 0.012 | - | Stn 101 higher *f*CDOM |
| **NO_3_** | - | - | 0.116 | ns |
| **NO_2_** | - | - | 0.014 | Stn 115 higher NO_2_ |
| **Si** | - | - | 0.464 | ns |
| **PO_4_** | - | - | 9.00E-03 | Stn 101 higher PO_4_ |
| **Si:N** | - | - | 0.174 | ns |

**Table S3**: Relative abundance of taxa in small and large fractions from the DNA template, based on OTUs at the lowest reliable taxonomic level with occurrences of >1% of the overall reads in at least one DNA sample. Values less than 0.1% indicated as a dash (-).

|  |  | **Station 101** | | | | **Station 115** | | | |
| --- | --- | --- | --- | --- | --- | --- | --- | --- | --- |
|  |  | **T1 Small** | **T1 Large** | **T4 Small** | **T4 Large** | **T1 Small** | **T1 Large** | **T4 Small** | **T4 Large** |
| **Ciliophora** | *Oligotrichida* | 0.140 | 0.057 | 0.221 | 0.030 | 0.238 | 0.044 | 0.086 | 0.025 |
|  | *Choreotrichida* | 0.023 | 0.007 | 0.051 | 0.009 | 0.016 | 0.007 | 0.015 | 0.007 |
|  | *Askenasia* | 0.015 | 0.003 | 0.018 | 0.005 | 0.001 | - | 0.001 | - |
|  | *Other* | 0.010 | 0.002 | 0.019 | 0.005 | 0.004 | 0.002 | 0.011 | 0.002 |
|  | **TOTAL Ciliophora** | **0.188** | **0.069** | **0.310** | **0.049** | **0.259** | **0.053** | **0.113** | **0.034** |
| **Dinoflagellates** | *Other* | 0.225 | 0.191 | 0.183 | 0.160 | 0.141 | 0.160 | 0.268 | 0.251 |
|  | *Gyrodinium sp.* | 0.045 | 0.267 | 0.098 | 0.483 | 0.062 | 0.151 | 0.103 | 0.197 |
|  | Gymnodiniales | 0.022 | 0.048 | 0.027 | 0.068 | 0.027 | 0.062 | 0.116 | 0.132 |
|  | *Nematodinium* | 0.022 | 0.056 | 0.011 | 0.052 | 0.035 | 0.121 | 0.030 | 0.153 |
|  | *Gyrodinium spirale* | 0.002 | 0.034 | 0.001 | 0.028 | 0.001 | 0.002 | 0.002 | 0.007 |
|  | *Gyrodinium clone AN0630L10* | 0.006 | 0.102 | 0.001 | 0.019 | 0.001 | - | - | 0.003 |
|  | *Gyrodinium helveticum* | 0.003 | 0.013 | 0.006 | 0.035 | 0.003 | 0.009 | 0.007 | 0.012 |
|  | **TOTAL Dinoflagellates** | **0.325** | **0.711** | **0.327** | **0.844** | **0.269** | **0.504** | **0.526** | **0.755** |
| **MALVs** | *MALV I [Duboscquellida]* | 0.008 | 0.014 | 0.005 | 0.010 | 0.049 | 0.016 | 0.044 | 0.044 |
|  | *MALV III [Syndinales]* | 0.005 | 0.002 | 0.011 | 0.003 | 0.013 | 0.010 | 0.010 | 0.004 |
| **Haptophyceae** | *Chrysochromulina* | 0.014 | 0.006 | 0.001 | 0.002 | 0.002 | 0.002 | 0.003 | 0.002 |
| **Other** | *Other Alveolata* | 0.001 | - | 0.003 | 0.001 | 0.027 | 0.015 | 0.007 | 0.003 |
| **Picozoa** | *Uncultured Arctic clone NW617,02* | 0.063 | 0.005 | 0.008 | 0.001 | 0.053 | 0.007 | 0.011 | 0.003 |
| **Chrysophyceae** | *Unclassified* | 0.151 | 0.086 | 0.077 | 0.019 | 0.001 | 0.003 | 0.004 | 0.004 |
|  | *Other Chrysophyceae* | 0.001 | - | 0.001 | - | 0.023 | 0.024 | 0.006 | 0.015 |
|  | *Unclassified clone FV18 1B10* | 0.033 | 0.016 | 0.016 | 0.005 | - | - | 0.001 | 0.001 |
| **Diatoms** | *Pseudo-nitzschia sp.* | - | - | - | 0.001 | 0.041 | 0.244 | 0.001 | 0.009 |
|  | *Chaetoceros gelidus* | 0.003 | 0.012 | - | 0.007 | 0.002 | 0.016 | 0.002 | 0.001 |
|  | *Chaetoceros neogracile* | 0.001 | 0.008 | - | 0.004 | 0.005 | 0.024 | 0.002 | 0.009 |
|  | **TOTAL Diatoms** | **0.004** | **0.020** | **0.001** | **0.011** | **0.048** | **0.284** | **0.005** | **0.019** |
| **MASTs** | *MAST 2 DH148 5 EKD52* | 0.002 | - | 0.001 | - | 0.039 | 0.009 | 0.014 | 0.004 |
| **Chlorophytes** | *Micromonas CCMP2099* | 0.044 | 0.001 | 0.122 | 0.004 | 0.018 | - | 0.040 | 0.002 |
|  | *Pyramimonas* | - | - | 0.001 | - | 0.013 | 0.002 | 0.049 | 0.010 |
|  | **TOTAL** | **0.841** | **0.930** | **0.883** | **0.950** | **0.814** | **0.928** | **0.832** | **0.899** |

**Table S4**: Operational taxonomic Units (OTUs) with > 1% of the reads in at least one sample. Relative abundance of taxa in small and large fractions based on OTUs at the lowest reliable taxonomic level with occurrences of  >1% of the overall reads in at least one RNA sample. Values less than 0.1% indicated as a dash (-). Average and standard deviation (Avg (sd)) **A)** Canadian side Stn 101 **B)** Greenland side Stn 115.

**A)**

| **Canadian** |  | **Stn 101 small** | | | | | | **Stn 101 large** | | | | | |
| --- | --- | --- | --- | --- | --- | --- | --- | --- | --- | --- | --- | --- | --- |
| **Major group** | **Taxon** | **T1** | **T2** | **T4** | **T5** | **T7** | **Avg (sd)** | **T1** | **T2** | **T4** | **T5** | **T7** | **Avg (sd)** |
| **Ciliophora** | Oligotrichida | 4.4 | 17.4 | 21.4 | 31.8 | 30.9 | 21.2 (11.2) | 9.6 | 10.3 | 12.3 | 24.4 | 23.7 | 16.0 (7.4) |
|  | Choreotrichida | 1.4 | 1.1 | 2.4 | 1.6 | 1.1 | 1.5 (0.5) | 1.6 | 3.1 | 3.2 | 2.2 | 3.2 | 2.7 (0.7) |
|  | *Askenasia* | - | 0.2 | 2.7 | 1.2 | 1.8 | 1.2 (1.1) | 13.7 | 7.6 | 13.3 | 2.5 | 2.9 | 8.0 (5.4) |
|  | *Urotricha* | - | - | 0.2 | 0.9 | 0.6 | 0.3 (0.4) | 0.3 | 5.0 | 4.6 | 1.1 | 0.4 | 2.3 (2.3) |
|  | *Monodinium* | - | - | 0.7 | 0.1 | - | 0.2 (0.3) | - | 0.3 | 0.4 | 0.1 | - | 0.2 (0.2) |
|  | Litostomatea | 3.3 | 0.9 | 3.0 | 0.3 | 1.1 | 1.7 (1.4) | 2.4 | 0.1 | 0.3 | 0.1 | 0.4 | 0.7 (1.0) |
|  | Other Ciliophora | - | 0.3 | 0.7 | 0.6 | 0.6 | 0.5 (0.3) | 0.5 | 1.5 | 1.2 | 0.5 | 0.8 | 0.9 (0.5) |
| **Dinoflagellates** | *Gyrodinium spirale* | 0.1 | 0.7 | 7.3 | 2.6 | 3.1 | 2.7 (2.8) | 1.0 | 2.4 | 1.0 | 3.2 | 2.9 | 2.1 (1.1) |
|  | *Gyrodinium* sp. | 0.7 | 1.9 | 16.9 | 13.6 | 12.8 | 9.2 (7.4) | 3.7 | 8.5 | 3.5 | 14.0 | 12.1 | 8.3 (4.8) |
|  | *Gymnodiniales* | 0.2 | 0.7 | 3.0 | 1.9 | 2.2 | 1.6 (1.1) | 1.4 | 2.6 | 1.9 | 2.4 | 1.9 | 2.0 (0.5) |
|  | *Nematodinium* | 0.2 | 0.8 | 0.9 | 1.9 | 1.9 | 1.2 (0.8) | 2.1 | 1.8 | 1.6 | 2.0 | 1.3 | 1.7 (0.3) |
|  | Other Dino | 2.8 | 15.6 | 12.4 | 15.0 | 17.9 | 12.8 (5.9) | 19.9 | 20.5 | 17.1 | 14.4 | 21.5 | 18.7 (2.9) |
| **Syndinales** | Duboscquellida | 0.1 | 0.1 | - | 0.1 | 0.1 | 0.1 (-) | 0.1 | 0.2 | 0.3 | 0.1 | 0.2 | 0.2 (0.1) |
|  | MALV III | - | 1.6 | 0.1 | 1.6 | 1.3 | 0.9 (0.8) | 1.2 | 1.3 | 2.2 | 1.6 | 1.0 | 1.4 (0.4) |
| **Chrysophyceae** | Other Chrysophyceae | - | 0.2 | 0.1 | - | - | 0.1 (0.1) | 0.3 | 0.5 | 0.3 | 0.2 | 0.4 | 0.4 (0.1) |
|  | Unclassified clone FV18 1B10 | - | 0.5 | 2.1 | 1.0 | 0.4 | 0.8 (0.8) | 7.3 | 4.4 | 5.8 | 3.5 | 2.0 | 4.6 (2.0) |
|  | Unclassified Chryso | - | 1.9 | 7.9 | 4.2 | 1.9 | 3.2 (3.0) | 26.5 | 18.6 | 22.6 | 14.5 | 8.8 | 18.2 (6.9) |
| **Diatoms** | *Pseudo-nitzschia* spp. | 0.2 | 0.1 | - | - | - | 0.1 (0.1) | 0.1 | - | - | - | - | - |
|  | *Chaetoceros neogracile* | - | 0.1 | - | - | - | - | 0.3 | 0.3 | 0.1 | 0.6 | 1.2 | 0.5 (0.4) |
|  | *Chaetoceros gelidus* | 72.5 | 1.4 | 6.6 | - | - | 16.1 (31.6) | 0.3 | 0.2 | 0.1 | - | 0.3 | 0.2 (0.1) |
| **Haptophyceae** | *Phaeocystis* | 0.9 | 42.9 | 0.4 | - | - | 8.8 (19.1) | - | - | - | - | 0.1 | - |
|  | *Chrysochromulina* | 0.6 | 0.2 | 2.5 | 0.3 | 0.5 | 0.8 (1.0) | 0.4 | 1.0 | 0.2 | 1.2 | 3.3 | 1.2 (1.2) |
| **Chlorophytes** | *Micromonas CCMP2099* | 1.4 | 0.6 | - | 12.5 | 15.7 | 6.1 (7.5) | - | 0.2 | 0.1 | 0.7 | 0.8 | 0.4 (0.4) |
|  | *Pyramimonas* | 0.1 | - | - | 0.5 | - | 0.1 (0.2) | - | 0.1 | - | 0.2 | - | 0.1 (0.1) |
| **Picozoa** | *Arctic clone NW617,02* | 0.5 | 0.8 | 0.3 | 0.1 | 0.3 | 0.4 (0.3) | - | - | - | 0.1 | 0.2 | 0.1 (0.1) |
| **MASTs** | *MAST 2 DH148 5 EKD52* | - | 1.3 | - | - | - | 0.3 (0.6) | - | - | - | - | - | - |
|  | **Total abundant taxa** | **89.6** | **91.1** | **91.7** | **92.0** | **94.2** | **91.7** | **93.6** | **90.9** | **92.4** | **89.9** | **89.2** | **91.2** |

**B)**

| **Greenland** |  | **S115 small** | | | | | | **S115 large** | | | | | | |
| --- | --- | --- | --- | --- | --- | --- | --- | --- | --- | --- | --- | --- | --- | --- |
| **Major group** | **Taxon** | **T1** | **T2** | **T4** | **T5** | **T7** | **Avg (sd)** | | **T1** | **T2** | **T4** | **T5** | **T7** | **Avg (sd)** |
| **Ciliophora** | Oligotrichida | 43.1 | 33.6 | 31.5 | 51.3 | 30.7 | 38.1 (8.9) | | 3.8 | 6.2 | 8.9 | 10.2 | 5.4 | 6.9 (2.6) |
|  | Choreotrichida | 1.0 | 0.4 | 1.3 | 1.1 | 0.8 | 0.9 (0.4) | | 1.1 | 0.8 | 5.1 | 0.9 | 0.6 | 1.7 (1.9) |
|  | *Askenasia* | 0.2 | 0.3 | 0.5 | 8.2 | 0.5 | 2.0 (3.5) | | 0.1 | 0.3 | 0.6 | 3.5 | 0.8 | 1.1 (1.4) |
|  | *Urotricha* | 0.1 | - | 0.3 | 0.1 | - | 0.1 (0.1) | | - | 0.1 | 0.4 | - | - | 0.1 (0.2) |
|  | *Monodinium* | 0.2 | 0.4 | 10.7 | 0.3 | 0.5 | 2.4 (4.6) | | 0.1 | 0.7 | 5.1 | 0.1 | 0.3 | 1.3 (2.2) |
|  | Litostomatea | 2.6 | 2.3 | 3.8 | 1.7 | 1.1 | 2.3 (1.0) | | 0.8 | 1.0 | 0.8 | 0.1 | 0.2 | 0.6 (0.4) |
|  | Other Ciliophora | 0.3 | 0.3 | 1.7 | 1.3 | 1.5 | 1.0 (0.7) | | 0.2 | 0.6 | 6.7 | 0.9 | 4.6 | 2.6 (2.9) |
| **Dinoflagellates** | *Gyrodinium spirale* | 0.3 | 0.3 | 0.4 | 0.4 | 0.6 | 0.4 (0.1) | | - | 0.1 | 0.1 | 0.2 | 0.1 | 0.1 (0.1) |
|  | *Gyrodinium* sp. | 2.3 | 2.0 | 2.3 | 1.5 | 4.2 | 2.5 (1.0) | | 0.7 | 1.2 | 1.0 | 0.9 | 0.5 | 0.9 (0.3) |
|  | *Gymnodiniales* | 1.1 | 1.5 | 4.6 | 1.6 | 2.5 | 2.3 (1.4) | | 1.1 | 2.0 | 3.5 | 2.0 | 1.3 | 2.0 (1.0) |
|  | *Nematodinium* | 2.0 | 1.5 | 2.1 | 1.2 | 2.5 | 1.9 (0.5) | | 1.5 | 1.7 | 1.8 | 1.2 | 0.9 | 1.4 (0.4) |
|  | Other Dino | 12.0 | 16.0 | 18.2 | 10.4 | 18.7 | 15.1 (3.7) | | 11.5 | 19.2 | 21.4 | 16.3 | 11.4 | 16.0 (4.5) |
| **Syndinales** | Duboscquellida | 0.9 | 0.7 | 0.9 | 0.8 | 0.6 | 0.8 (0.1) | | 1.0 | 1.3 | 3.6 | 1.3 | 1.2 | 1.7 (1.1) |
|  | MALV III | 3.5 | 8.5 | 2.8 | 0.8 | 5.0 | 4.1 (2.9) | | 1.9 | 3.6 | 2.5 | 0.6 | 1.9 | 2.1 (1.1) |
| **Chrysophyceae** | Other Chrysophyceae | 1.9 | 3.8 | 0.7 | 3.8 | 2.3 | 2.5 (1.3) | | 13.6 | 17.0 | 13.2 | 19.0 | 10.9 | 14.7 (3.2) |
|  | Unclassified clone FV18 1B10 | - | - | 0.1 | - | 0.3 | 0.1 (0.1) | | 0.1 | 0.1 | 0.9 | 0.2 | 0.9 | 0.4 (0.4) |
|  | Unclassified Chryso | 0.2 | 0.5 | 0.6 | 0.3 | 1.5 | 0.6 (0.5) | | 2.0 | 2.5 | 5.4 | 3.5 | 6.1 | 3.9 (1.8) |
| **Diatoms** | *Pseudo-nitzschia spp.* | 0.6 | 0.1 | - | 1.0 | 0.4 | 0.4 (0.4) | | 51.9 | 32.1 | 4.0 | 27.7 | 43.0 | 31.7 (18.2) |
|  | *Chaetoceros neogracile* | 0.2 | 0.1 | - | 0.6 | 0.1 | 0.2 (0.3) | | 1.0 | 0.4 | 0.5 | 2.5 | 0.8 | 1.0 (0.9) |
|  | *Chaetoceros gelidus* | - | - | - | 0.1 | - | - | | 1.1 | 0.2 | - | 1.1 | 0.9 | 0.7 (0.5) |
| **Haptophyceae** | *Phaeocystis* | 0.5 | 0.3 | 0.3 | 0.3 | 1.1 | 0.5 (0.4) | | 0.1 | - | - | 0.1 | 0.2 | 0.1 (0.1) |
|  | *Chrysochromulina* | 0.7 | 1.0 | 0.6 | 0.3 | 0.9 | 0.7 (0.3) | | 0.1 | 0.1 | 0.2 | 0.2 | 0.4 | 0.2 (0.1) |
| **Chlorophytes** | *Micromonas CCMP2099* | 2.8 | 4.8 | 2.1 | 2.7 | 7.0 | 3.8 (2.0) | | - | - | - | - | - | - |
|  | *Pyramimonas* | 2.6 | 2.7 | 3.6 | 0.9 | 4.7 | 2.9 (1.4) | | 0.1 | 0.2 | 1.2 | 0.4 | 0.4 | 0.5 (0.4) |
| **Picozoa** | *Arctic clone NW617,02* | 6.3 | 2.7 | 0.1 | 0.1 | 1.3 | 2.1 (2.6) | | - | - | - | - | 0.1 | - |
| **MASTs** | *MAST 2 DH148 5 EKD52* | 3.2 | 3.9 | 0.5 | 1.4 | 0.5 | 1.9 (1.6) | | 0.6 | 0.7 | 0.2 | 0.5 | 0.1 | 0.4 (0.3) |
|  | **Total abundant taxa** | **88.6** | **87.9** | **89.8** | **92.2** | **89.4** | **89.6** | | **94.3** | **92.1** | **87.9** | **93.6** | **93.1** | **92.2** |
|  |  |  |  |  |  |  |  | |  |  |  |  |  |  |

**Table S5**: Bray-Curtis similarity of communities from the two sides of the North Water. Bray-Curtis distances as % similarity of communities from the Canadian (Stn 101), Greenland (Stn 115) sides. **A)** The five 20 m small fraction communities. **B)** The five 20 m large fraction communities.

| **A)** | **Small Fraction: Bray-Curtis similarity index** | | | | |
| --- | --- | --- | --- | --- | --- |
| **Stn 101** | 20 m (T_1_) | 20 m (T_2_) | 20 m (T_4_) | 20 m (T_5_) | 20 m (T_7_) |
| 20m (T_1_) | – |  |  |  |  |
| 20m (T_2_) | 21% | – |  |  |  |
| 20m (T_4_) | 25% | 51% | – |  |  |
| 20m (T_5_) | 17% | 50% | 77% | – |  |
| 20m (T_7_) | 17% | 49% | 77% | 94% | – |
| **Stn 115** | 20 m (T_2_) | 20 m (T_1_) | 20 m (T_4_) | 20 m (T_5_) | 20 m (T_7_) |
| 20m (T_2_) | – |  |  |  |  |
| 20m (T_1_) | 85% | – |  |  |  |
| 20m (T_4_) | 80% | 85% | – |  |  |
| 20m (T_5_) | 73% | 82% | 81% | – |  |
| 20m (T_7_) | 86% | 76% | 85% | 69% | – |
| **B)** | **Large fraction: Bray-Curtis similarity index** | | | | |
| **Stn 101** | 20 m (T_1_) | 20 m (T_2_) | 20 m (T_4_) | 20 m (T_5_) | 20 m (T_7_) |
| 20m (T_1_) | – |  |  |  |  |
| 20m (T_2_) | 88% | – |  |  |  |
| 20m (T_4_) | 90% | 86% | – |  |  |
| 20m (T_5_) | 82% | 92% | 87% | – |  |
| 20m (T_7_) | 77% | 87% | 78% | 90% | – |
| **Stn 115** | 20 m (T_1_) | 20 m (T_2_) | 20 m (T_4_) | 20 m (T_5_) | 20 m (T_7_) |
| 20m (T_1_) | – |  |  |  |  |
| 20m (T_2_) | 79% | – |  |  |  |
| 20m (T_4_) | 51% | 72% | – |  |  |
| 20m (T_5_) | 75% | 90% | 71% | – |  |
| 20m (T_7_) | 90% | 84% | 60% | 84% | – |


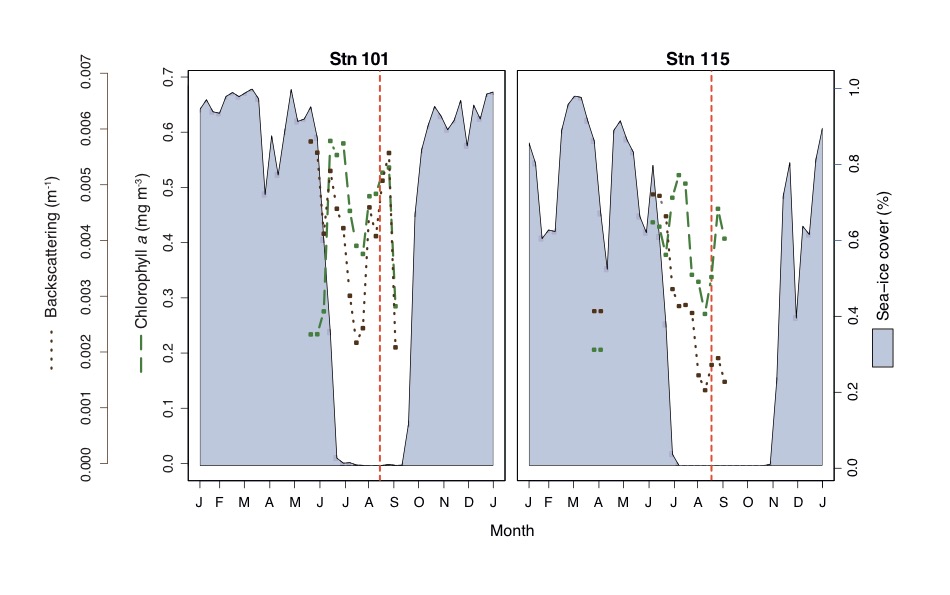
**Figure S1:** Composite satellite data**.** Sea ice cover, chlorophyll *a* concentration and particulate back-scattering coefficient at 443 nm estimated from satellite data. Stn 101 (Canada) and Stn 115 (Greenland). Vertical dashed lines are the date of sampling; 15 August 2013 for Stn 101 and 18 August 2013 for Stn 115. Shaded area is % sea ice cover.


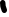


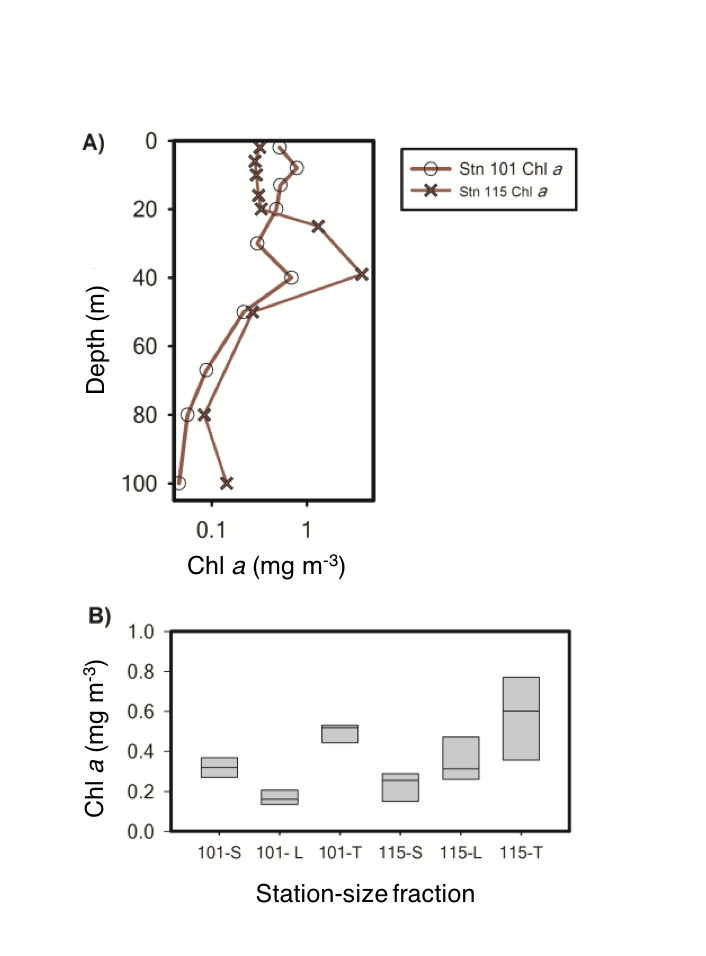


**Figure S2**: Extracted chlorophyll from the two sides of the North water. **A)** Profiles of total Chl *a* from the Canadian (Stn 101) and Greenland (Stn 115). Stn 101 samples collected at T_1_ and Stn 115 at T_2_ (**Table S1**). **B)** Size fractionated Chl *a* concentrations: smaller fraction (S) from 0.8–5 µm, larger (L) fraction > 5 µm and total (T***)*.** Box plots (n = 7) of repeated sampling from 20 m at the two stations. Central line is the median; differences between the stations were not significant (ANOVA repeated measures).


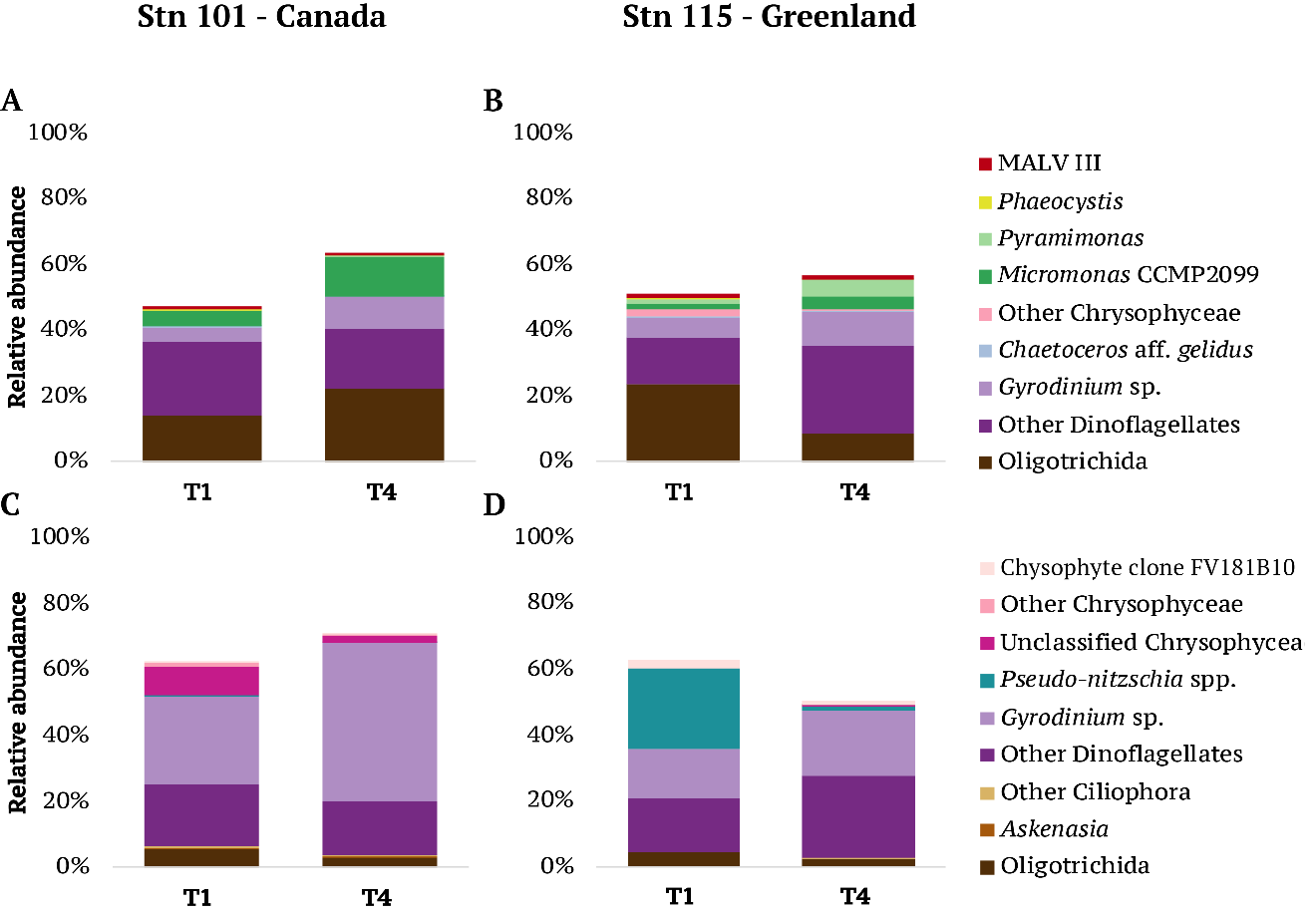


**Figure S3**: Barplot using DNA samples showing the most abundant species from the RNA. DNA was sequenced from two time points (T_1_ and T_4_) on the Canada side (A and C) and Greenland side (B and D) of Northern Baffin Bay for small (A and B) and large (C and D) fractions.

**
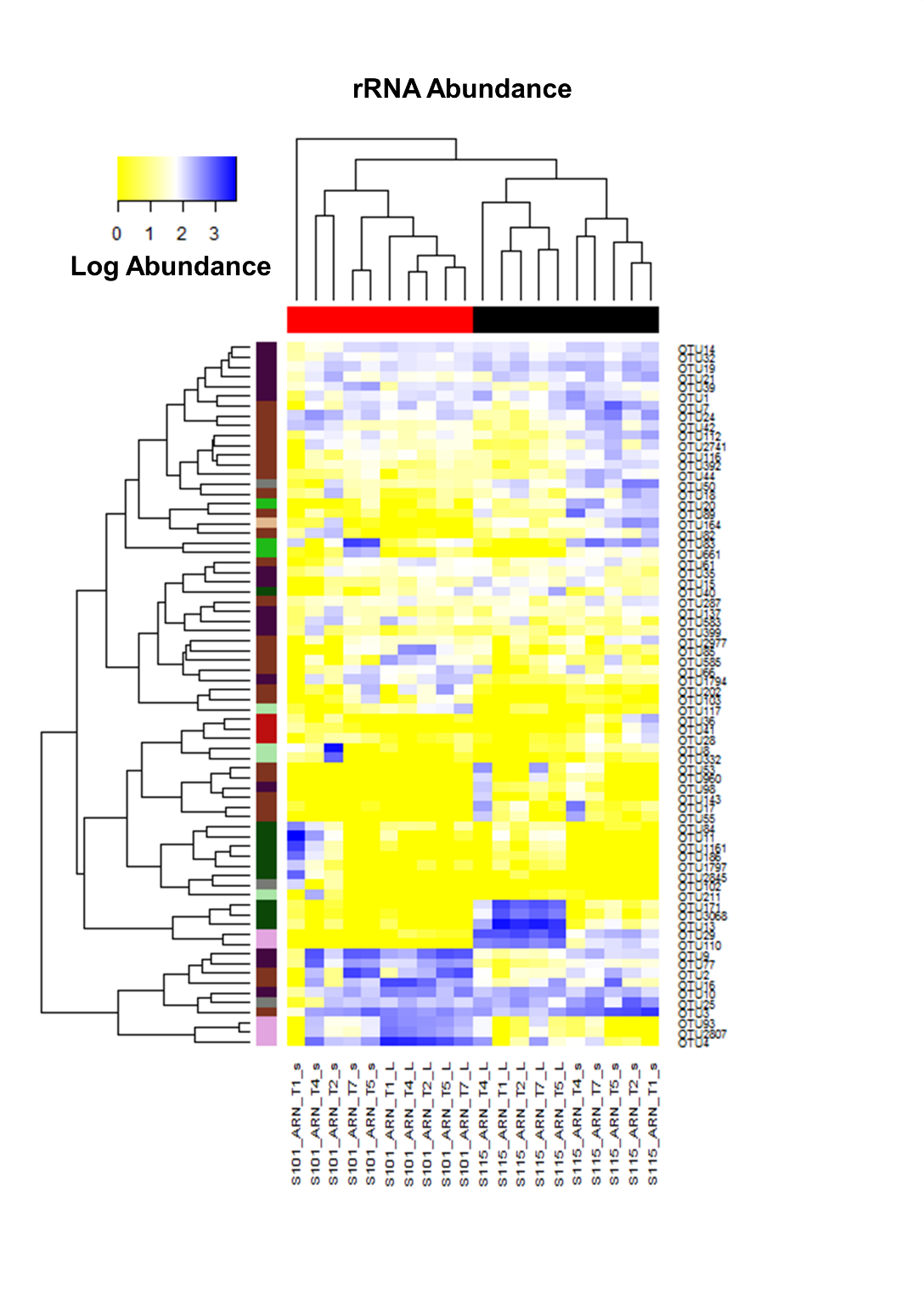
**

**Figure S4**: Heatmap from the reads of OTUs from rRNA accounting for at least 100 occurrences in one RNA 20 m sample from both sides. OTUs are in columns, color coded for taxonomic groups. Samples labels indicate station, time collected and small (S, 0.2-3 µm), or large (L, 3-50 µm) fractions. Color code as for Figure S3. Identity of OTUs available at: <https://zenodo.org/record/1205261#.WrOAMJPwb-Y>.

**A)**


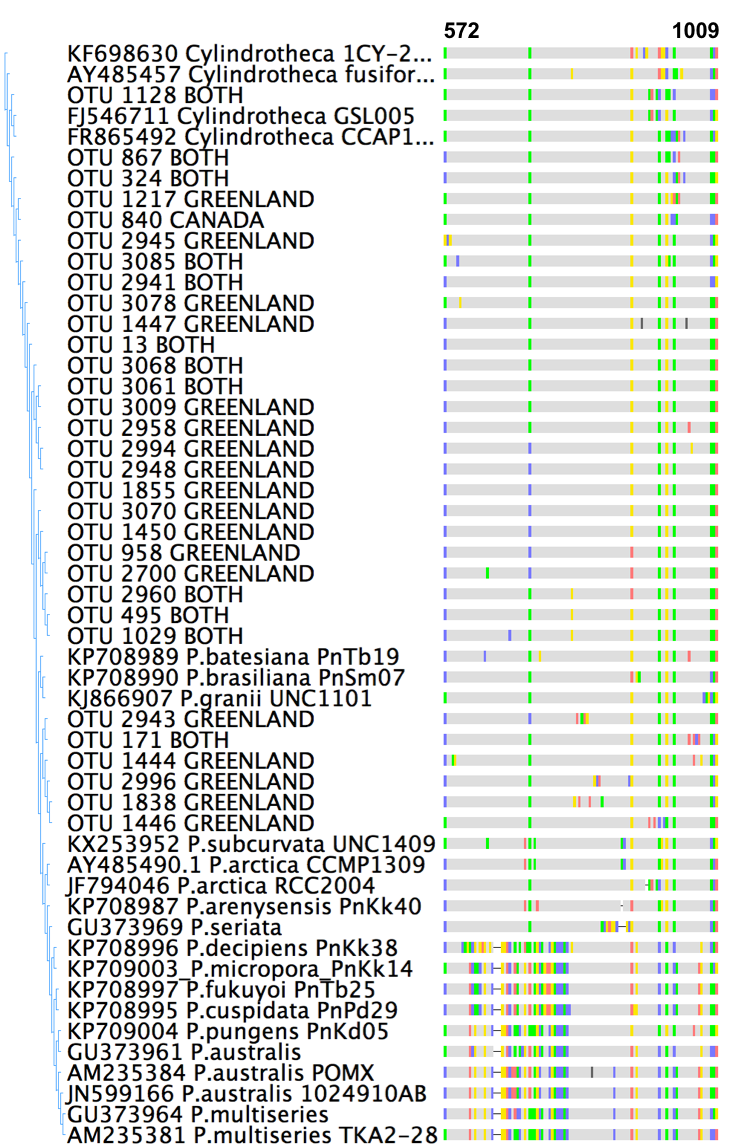


**B)**


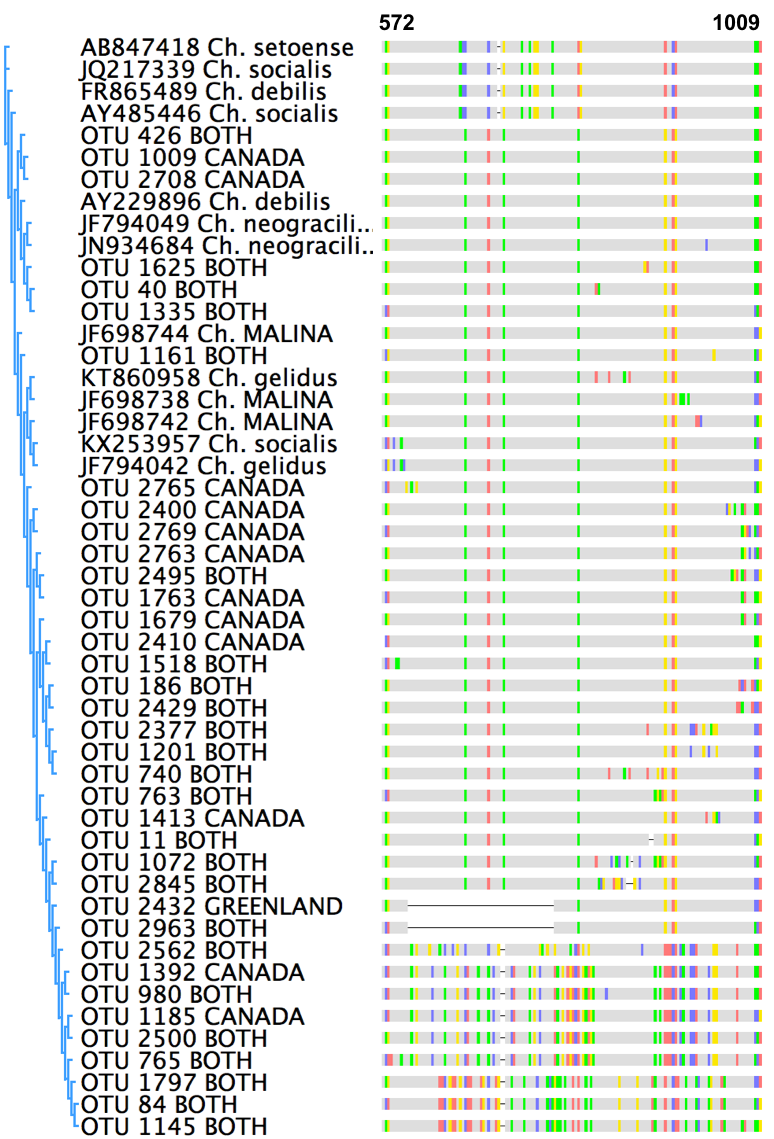


**Figure S5**: V4 region alignments. Position 572nt to 1009nt of the 18S rRNA gene. Reference sequences, with GenBank accessions and individual OTUs found on the GREENLAND, CANADA or BOTH sides. For visual comfort, identical bases are masked **A)** *Pseudo-nitzschia* and *Cylindrothica* (the closest related genus). The break between the genera is between OTU2945 (*Cylindrotheca*) and OTU3085 (*Pseudo-nitzschia*). **B)** Hylochaete *Chaetoceros*.


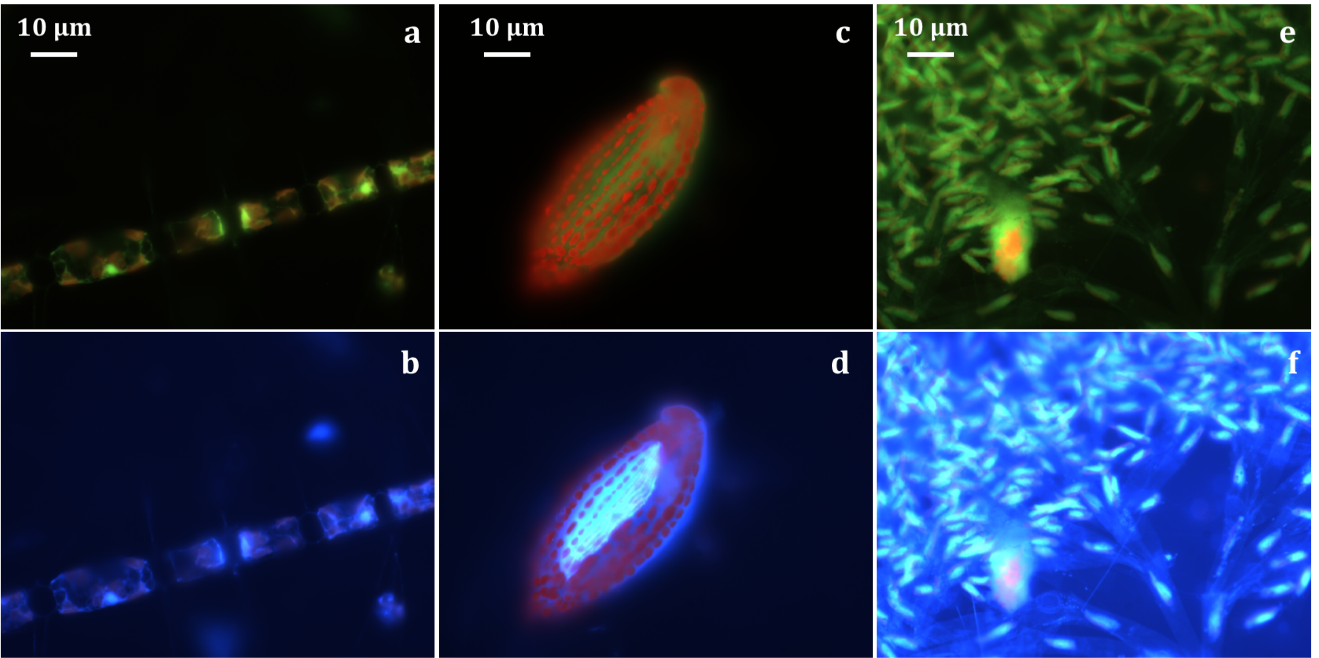


**
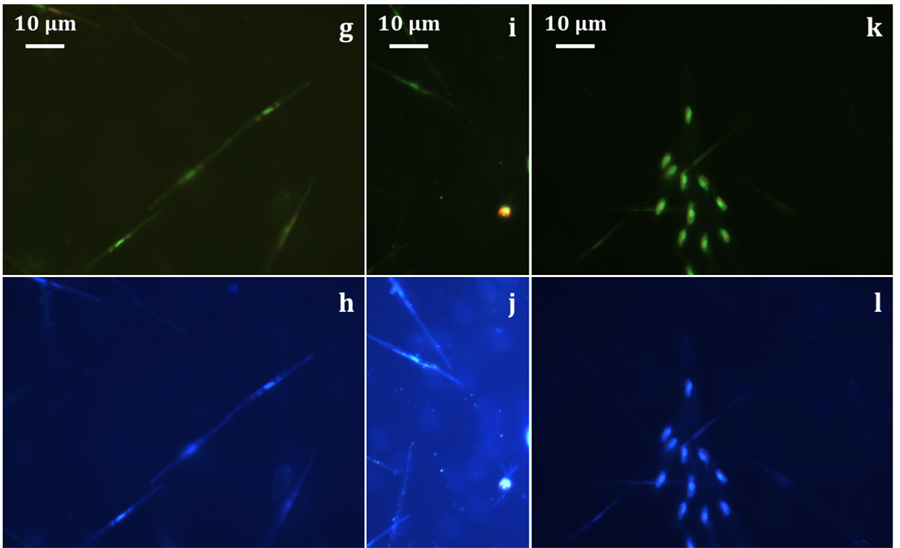
**

**Figure S6**: Epifluorescence micrographs showing DAPI stained cells under blue (a,c,e,g,i,k) and UV (b,d,f,h,j,l) excitation. Images from samples collected at Stn 101 with (a,b) *Chaetoceros gelidus*, (c,d) *Torodinium robustum*, and (e,f) *Dinobryon balticum*. Images from samples collected at Stn 115 with (g,h) *Pseudo-nitzschia* spp., (i,j) *Pyramimonas*, and (k,l) *Dinobryon belgica*.
